# N-acetylaspartate catabolism determines cytosolic acetyl-CoA levels and histone acetylation in brown adipocytes

**DOI:** 10.1038/srep23723

**Published:** 2016-04-05

**Authors:** A. Prokesch, H. J. Pelzmann, A. R. Pessentheiner, K. Huber, C. T. Madreiter-Sokolowski, A. Drougard, M. Schittmayer, D. Kolb, C. Magnes, G. Trausinger, W. F. Graier, R. Birner-Gruenberger, J. A. Pospisilik, J. G. Bogner-Strauss

**Affiliations:** 1 Institute of Biochemistry, Graz University of Technology, Austria; 2 Institute of Cell Biology, Histology and Embryology, Medical University of Graz, Austria; 3 Institute of Molecular Biology and Biochemistry, Medical University of Graz, Austria; 4 Research Unit Functional Proteomics and Metabolic Pathways, Institute of Pathology, Medical University of Graz and Omics Center Graz, BioTechMed-Graz, Austria; 5 ZMF, Center for Medical Research, Medical University of Graz, Austria; 6 HEALTH Insitute for Biomedicine and Health Sciences, Joanneum Research, Graz, Austria; 7 Max Planck Institute of Immunobiology and Epigenetics, Freiburg, Germany

## Abstract

Histone acetylation depends on the abundance of nucleo-cytoplasmic acetyl-CoA. Here, we present a novel route for cytoplasmic acetyl-CoA production in brown adipocytes. N-acetylaspartate (NAA) is a highly abundant brain metabolite catabolized by aspartoacylase yielding aspartate and acetate. The latter can be further used for acetyl-CoA production. Prior to this work, the presence of NAA has not been described in adipocytes. Here, we show that accumulation of NAA decreases the brown adipocyte phenotype. We increased intracellular NAA concentrations in brown adipocytes via media supplementation or knock-down of aspartoacylase and measured reduced lipolysis, thermogenic gene expression, and oxygen consumption. Combinations of approaches to increase intracellular NAA levels showed additive effects on lipolysis and gene repression, nearly abolishing the expression of Ucp1, Cidea, Prdm16, and Ppara. Transcriptome analyses of aspartoacylase knock-down cells indicate deficiencies in acetyl-CoA and lipid metabolism. Concordantly, cytoplasmic acetyl-CoA levels and global histone H3 acetylation were decreased. Further, activating histone marks (H3K27ac and H3K9ac) in promoters/enhancers of brown marker genes showed reduced acetylation status. Taken together, we present a novel route for cytoplasmic acetyl-CoA production in brown adipocytes. Thereby, we mechanistically connect the NAA pathway to the epigenomic regulation of gene expression, modulating the phenotype of brown adipocytes.

In contrast to white adipocytes that store energy as triglycerides in uni-locular lipid droplets, brown adipocytes exhibit multi-locular lipid droplets, are rich in mitochondria, and evolved to dissipate energy as heat in endothermal, placental animals^1^,^2^,^3^. Mechanistically, energy dissipation is achieved by its most prominent molecular marker, uncoupling protein-1 (Ucp1)^4^. Located in the inner mitochondrial membrane, Ucp1 diminishes the proton gradient built up by the electron transport chain thereby uncoupling cellular respiration from ATP synthesis. The discovery of functional brown adipose tissue (BAT) in adult humans^5^,^6^,^7^,^8^,^9^ opened the possibility that activation of BAT and “browning” of white adipose tissue could serve as therapeutic measure to control the obesity epidemic^10^. The classical activators of thermogenic, energy-dissipating activity in BAT are cold, catecholamines and thyroid hormone, all of which have severe limitations in clinical practice and/or serious side effects^11^. Hence, there is an ongoing search for alternative compounds and strategies that effectively and specifically activate BAT.

N-acetylaspartate (NAA) is the second most abundant metabolite in brain with concentrations ~10 mM^12^. In neurons, aspartate N-acetyltransferase (Asp-NAT, encoded by the gene Nat8l) produces NAA from acetyl-CoA and aspartate. NAA is then transported to oligodendrocytes where it is enzymatically cleaved by aspartoacylase (Aspa) to yield aspartate and acetate^13^. Subsequently, acetate is incorporated into acetyl-CoA^14^, which is a general energy metabolite and second messenger^15^, and acts as a precursor for lipid synthesis (i.e. myelination in brain^14^). The importance of an intact NAA pathway is apparent as deleterious mutations in the human ASPA gene lead to Canavan disease, a fatal neurological disorder caused by a myelination defect in the central nervous system^16^,^17^. This shows that the amount of NAA in brain is strictly regulated and imbalances of brain NAA impact development and behaviour in man and mice^12^,^18^,^19^,^20^,^21^. Patients with Canavan disease have highly increased brain NAA levels and show an excessive urinary NAA excretion^18^. Apart from Canavan disease, there is increasing evidence for a pivotal role of NAA in peripheral tissues and metabolic disorders^22^,^23^,^24^. We recently showed that expression of Nat8l is significantly reduced in white and brown adipose tissue of obese (ob/ob) mice, while it is unchanged in brain^25^. Additionally, urinary NAA levels are diminished in ob/ob mice^26^. Further, Nat8l expression is significantly decreased in subcutaneous fat of insulin-resistant obese humans when compared to insulin-sensitive obese humans^27^. On the contrary, urinary NAA levels increase upon aging in diabetic Zucker rats and are elevated in human diabetic patients with a body mass index between 25 and 40^28^. Notably, NAA is also available from exogenous sources as it is present in a variety of foods^29^. Studies with rats exhibited that dietary-supplemented NAA is bioavailable^30^, but neither evokes systemic toxicity nor adverse effects on the CNS^30^,^31^ as NAA does not cross the blood brain barrier^30^,^32^. Together, these data suggest a prominent physiological role of NAA also in peripheral tissues.

Our previous data showed that constitutive overexpression of Nat8l in immortalized brown adipocytes (iBACs) leads to increased lipid turnover, brown marker gene expression, mitochondrial biogenesis, and ultimately, mitochondrial respiration^25^. Additionally, stable overexpression of Nat8l in iBACs increased Aspa expression. These data led to the hypothesis that increased flux through the NAA pathway provides cytoplasmic acetyl-CoA. In this study we focused on the effects downstream of Nat8l. We increased intracellular NAA levels in brown adipocytes through either exogenous NAA treatment or stable knock-down of Aspa, all leading to reduced FFA release and brown marker gene expression. Mechanistically, we show that the observed transcriptional repression, resulting from elevated NAA levels after Aspa-knock-down, is accompanied by decreased cytoplasmic acetyl-CoA levels and histone lysine acetylation. In summary, our work connects the NAA pathway to the regulation of the brown adipocyte transcriptome and with that its phenotype.

## Results

### NAA treatment of iBACs reduces the brown adipocyte phenotype

**Figure 1:**
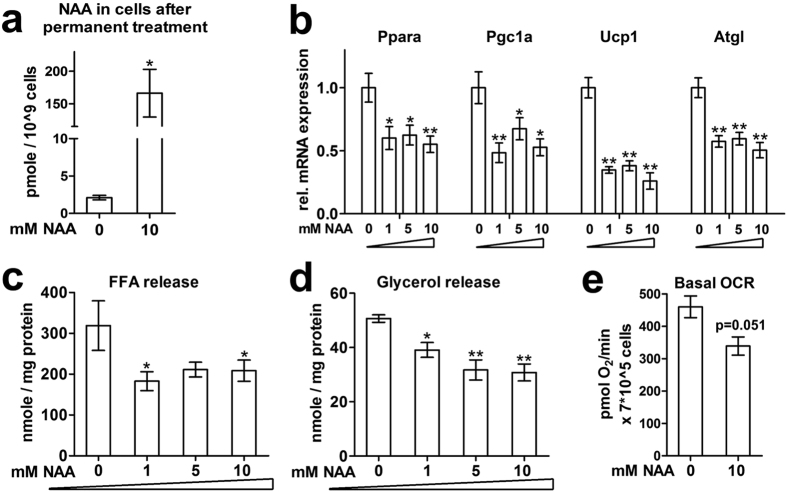
Long-term NAA treatment reduces brown adipocyte phenotype. (**a**) Immortalized brown adipocyte cells (iBACs) were differentiated for seven days with or without 10 mM NAA supplementation to the medium. Cells were washed, harvested, and prepared for LC-MS/MS to specifically detect NAA (174/88). (**b–d**) NAA was supplemented to the medium in varying concentrations throughout differentiation of iBACs. Measurements were performed on day 7 (n = 3). (**b**) mRNA levels of brown marker genes. (**c**) Free fatty acid (FFA) release into medium. (**d**) Glycerol release into medium. (**e**) Basal oxygen consumption rate measured with a Seahorse XF96 extracellular flux analyzer in d7 iBACs with or without NAA (n = 3). Student’s t-test compared to 0 mM NAA: *p < 0.05; **p < 0.01.

Recently, we showed that increased expression of Nat8l and Aspa enhances the phenotype of brown adipocytes^25^. We speculated that this might be due to increasing the flux through the NAA pathway. Here we tested the effects of exogenous NAA treatment. Since NAA transporters known to be expressed in brain (Slc13a1, Slc13a2, Slc13a3) are not detectable at the mRNA level in iBACs (data not shown), we first investigated if exogenously supplemented NAA enters these cells. Therefore, iBACs were differentiated in the absence or presence of 10 mM NAA-supplemented culture media. Using HPLC-MS/MS we showed a ~100-fold increase of intracellular NAA concentrations in iBACs differentiated with NAA (Fig. 1a). Next we treated differentiating iBACs with increasing concentrations (0, 1, 5, 10 mM) of NAA and evaluated the effect on brown adipocyte marker and key thermogenic gene expression in mature iBACs. Ppara, Pgc1a, Ucp1, and Atgl mRNA expression was already markedly reduced in the presence of 1mM NAA (Fig. 1b). As lipid droplet-derived FFAs serve as ligands for Ppara^33^ and as activators for Ucp1^34^, lipolysis is vital for brown adipocyte function^35^. Consistent with reduced brown marker gene expression, treatment of iBACs with NAA led to reduced lipolysis as shown by markedly reduced FFA (Fig. 1c) and glycerol release (Fig. 1d) into the medium. A prominent functional indicator of brown adipocyte activity is the degree of cellular respiration. Treatment of iBACs with 10 mM NAA reduced basal oxygen consumption rate (Fig. 1e). Together, decreased thermogenic gene expression, lipolysis, and oxygen consumption argue for a reduction of the brown phenotype in iBACs mediated by NAA supplementation.

### Aspa is highly expressed in brown adipocytes and silencing of Aspa leads to NAA accumulation and reduced brown adipocyte phenotype

**Figure 2:**
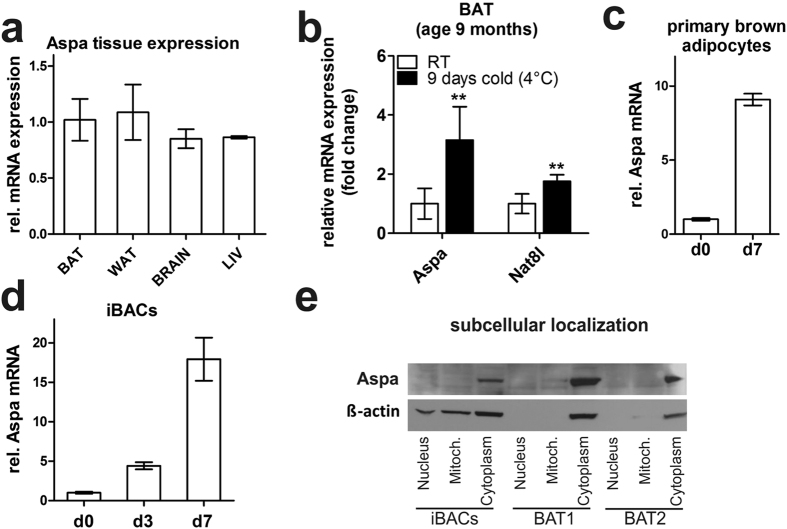
Aspa is expressed in brown adipose tissue and located to the cytoplasm in brown adipocytes. (**a**) Aspa tissue expression in 8–10 -week old, male, ad libitum-fed C57Bl/6 mice (n = 3). (**b**) Male C57Bl/6J mice (age 9 months) on chow diet were challenged by cold exposure (4–8 °C) for a period 9 consecutive days. (**c,d**) *In vitro* Aspa expression during differentiation of primary brown adipocytes (n = 2, (**c**)) and iBACs (n = 3, (**d**)). (**e**) Western blot after fractionation of mature iBACs and BAT from 8–10 weeks old, male, ad libitum-fed C57Bl/6 mice. Beta-actin serves as cytoplasmic control protein.

**Figure 3:**
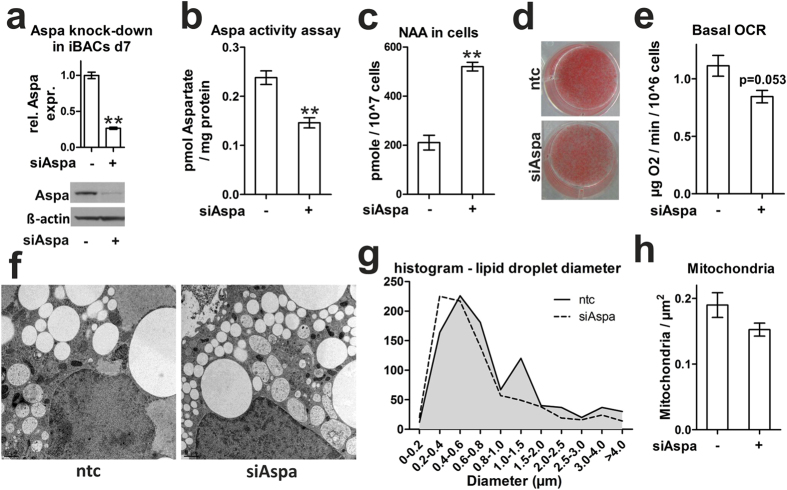
Knock-down of Aspa elevates intracellular NAA and changes ultrastructural features. (**a–h**) Aspa knock-down in iBACs. Undifferentiated iBACs were incubated with supernatant containing lentiviral Aspa shRNA (siAspa) or non-targeting control (ntc) followed by antibiotic selection. Subsequently stably silenced cells were differentiated for seven days and harvested. Student’s t-test (n = 3) compared to ntc: *p < 0.05; **p < 0.01. (**a**) Aspa mRNA levels with qPCR and immunoblot for Aspa protein levels. (**b**) Aspa activity assay was measured via fluorometric detection of aspartate. (**c**) Intracellular NAA concentrations measured using LC-MS/MS. (**d**) Oil red O staining of mature iBACs to visualize neutral lipids. (**e**) Basal oxygen consumption measured with a Clark electrode. (**f**) Electron micrographs of differentiated iBACs. Scale bar is 1 μm. (**g**) Diameters of more than 800 lipid droplets were analyzed from all siAspa and ntc electron micrographs. (**h**) Mitochondria were counted from 99 electron micrographs derived from three replicates and related to the area.

Aspa is described as the sole NAA-cleaving enzyme highly expressed in oligodendrocytes^36^. We show that Aspa is expressed in brown and white adipose tissue, and liver at levels comparable to whole brain lysates (Fig. 2a). Further, Aspa mRNA levels are induced in brown adipose tissue of cold-exposed mice (Fig. 2b) and strongly increase during differentiation of primary brown adipocytes (Fig. 2c) and iBACs (Fig. 2d). Aspa protein is located in the cytoplasm of fully differentiated iBACs and brown adipose tissue from mice (Fig. 2e), shown by western blot analysis after fractionation. To test whether forced reduction of Aspa expression leads to effects comparable to NAA treatment, we used a lentiviral shRNA-based approach to stably knock-down Aspa in iBACs. This strategy reduced Aspa mRNA and protein levels in differentiated iBACs by 80% (Fig. 3a) accompanied by a significant reduction of Aspa activity in knock-down cells (Fig. 3b). In accordance to the reported role of Aspa in brain as the sole NAA-catabolizing enzyme, NAA levels accumulated in Aspa knock-down iBACs compared to controls (Fig. 3c). Adipocyte differentiation capacity of iBACs was largely unaltered by Aspa knock-down, as shown by similar triglyceride accumulation (Fig. 3d). Indicative of attenuated brown adipocyte function, and possibly due to decreased number of mitochondria (Fig. 3f,h), basal oxygen consumption rate was reduced in Aspa knock-down cells (Fig. 3e). Additional analysis of electron micrographs showed overall smaller lipid droplets in Aspa knock-down cells (Fig. 3f,g).

### Knock-down of Aspa and concurrent NAA treatment reduce the brown phenotype of iBACs in an additive manner

**Figure 4:**
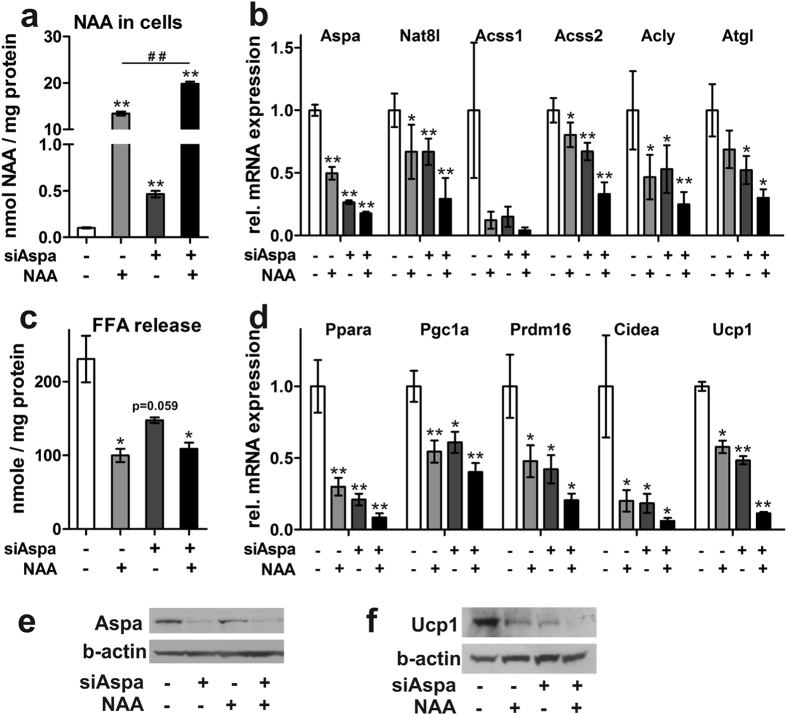
Aspa knock-down and elevated intracellular NAA levels reduce the brown phenotype in an additive manner. (**a–f**) iBACs with stable knock-down of Aspa or control cells were treated with 10 mM NAA throughout seven days of differentiation (n = 3). Normal differentiated cells served as a control. Student’s t-test (n = 3) compared to ntc: *p < 0.05; **p < 0.01; ***p > 0.001. (**a**) Metabolites were extracted by boiling cell lysates in ethanol after addition of internal standard mix and analysed with HPLC/HRMS. (**b**) mRNA levels of NAA pathway genes, mitochondrial acetyl-coA synthetase 1 (Acss1), cytoplasmic Acss2, ATP-citrate lyase (Acly), and adipose triglyceride lipase (Atgl). (**c**) Free fatty acid release into cell culture medium. (**d**) mRNA levels of brown marker genes. (**e,f**) Aspa and Ucp1 protein levels measured by immunoblot. Beta-actin serves as loading control.

Next, we differentiated Aspa knock-down cells with and without 10 mM NAA-supplemented to media. Intracellular NAA accumulates after Aspa knock-down or NAA treatment (Fig. 4a), and NAA levels show an additional significant increase after NAA supplementation to Aspa knock-down cells (Fig. 4a, black bar). Expression of members of the NAA pathway such as Nat8l, Aspa and acetyl-CoA synthase short-chain family member 1 and 2 (Acss1, Acss2) was reduced in individual treatments with the strongest repression upon combined treatment (see Fig. 4b for mRNA expression and 4e for Aspa protein expression). The same pattern was observed for Atgl (Fig. 4b) with a concomitant decrease in FFA release (Fig. 4c). In particular, NAA treatment of Aspaknock-down cells decreased the expression of brown/thermogenic marker genes Ppara, Pgc1a, Prdm16, Cidea, and Ucp1 to 10–40% of levels in untreated controls (black bars in Fig. 4d). Most importantly, Ucp1 protein abundance is strongly reduced after individual treatments and nearly abolished upon the combination of both (Fig. 4f). Together, additive repression of all measured transcripts was observed when exogenous NAA treatment and Aspa knock-down were combined (Fig. 4b,d), arguing for acting through a common pathway.

### Aspa knock-down leads to transcriptional repression via decreased cytoplasmic acetyl-CoA levels and reduction in histone lysine acetylation

**Figure 5:**
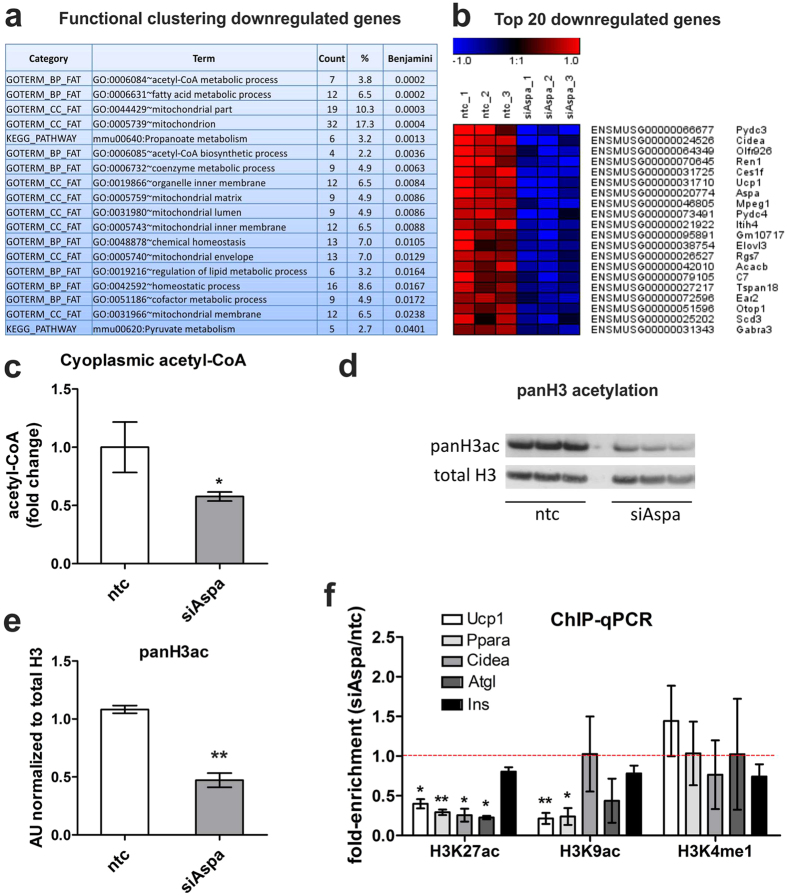
Aspa knock-down leads to global transcriptional repression, reduced cytoplasmic acetyl-CoA and histone acetylation. (**a–e**) Differentiated iBACs with stable knock-down of Aspa (siAspa) or non-targeting control cells (ntc) were used for Affymetrix microarray analysis, histone isolation, and ChIP-qPCR (each n = 3). (**a**) Two hundred and fifty genes downregulated by Aspa knock-down were submitted to DAVID functional annotation using GO terms and KEGG pathways. Count shows the number of mapped genes and Benjamini-Hochberg’s correction was used for adjusting p-value for multiple testing. (**b**) Top 20 genes significantly downregulated by Aspa knock-down. Ucp1 and Cidea are among the strongest repressed genes. (**c**) Acetyl-CoA measured with HPLC/HRMS in cytoplasmic fractions. Student’s t-test; *p < 0.05 (n = 3). (**d**) Western blot of histone preparations using an antibody against global histone H3 acetylation. Total histone H3 antibody serves as loading control. (**e**) Densitometric quantification of immunoblot. Student’s t-test; **p < 0.01. (**f**) ChIP-qPCR fold-enrichment of Aspa knock-down vs. control cells in promoters/enhancers of transcriptionally repressed genes. Histone acetylation was assessed on H3K27 and H3K9 using acetylation-specific antibodies for immunoprecipitation. Insulin (Ins) serves as non-regulated control region. The acetylation-independent activating histone mark H3K4me1 is not significantly changed. One-sample t-test; *p < 0.05; **p < 0.01.

Reduced mRNA levels of the measured transcripts following Aspa knock-down (Fig. 4b,d) suggests predominant transcriptional repression upon blockage of NAA catabolism. Indeed, comparison of genome-wide expression profiles from Aspa knock-down and control cells using microarrays showed a higher number of genes downregulated by Aspa knock-down (252 genes significantly down vs. 158 significantly up after Benjamini-Hochberg correction). Performing DAVID functional clustering on the set of genes upregulated by Aspa knock-down only yielded extracellular matrix-related gene ontology (GO) terms as significantly enriched (Supplemental Table 1). Performing the same analysis for genes downregulated by Aspa knock-down led to a significant enrichment of lipid metabolism-associated and mitochondria-related GO terms (Fig. 5a), also reflected in Ucp1 and Cidea among the highest ranking downregulated genes (Fig. 5b). Interestingly, highest ranking in this analysis is the acetyl-CoA metabolism process (GO:0006084, Fig. 5a). Most importantly, we also see a significant reduction of cytoplasmic acetyl-CoA levels (Fig. 5c) in Aspa-knock down cells when compared to controls. The acetyl moiety derived from acetyl-CoA is the sole substrate for histone acetyltransferases^15^ that are known to activate transcription by opening chromatin^37^. Hence, the observed constraint on cytoplasmic acetyl-CoA availability in Aspa knock-down cells (Fig. 5a,c) could be responsible for the transcriptional repression via changes in histone acetylation patterns. To substantiate this link, we performed western blot analysis on histone preparations from Aspa knock-down and control cells. We measured significantly reduced acetylation of histone H3 in Aspa knock-down cells (Fig. 5d,e for quantification). Acetylation of specific histone lysine residues has been shown to impact transcriptional regulation of nearby loci, with lysine-9 and lysine-27 acetylation as the best described marks for transcriptional activation^38^. To get more detailed insight, we performed chromatin immunoprecipitation with antibodies against specific histone modifications, followed by qPCR targeting promoters/enhancers of genes with significantly decreased expression after either Aspa knock-down and/or NAA treatment (Fig. 4b,d). We used antibodies against three histone modifications that are known to mark transcriptional active loci (H3K9ac, H3K27ac, H3K4me1). Interrogating enhancer or promoter regions of Ucp1, Ppara, Cidea, and Atgl, we see a strong reduction in the enrichment at all regions for H3K27ac and at most regions for H3K9ac (Fig. 5f) in Aspa knock-down cells. This is in agreement with the reduced gene expression levels shown in Fig. 4b,d. On the other hand, enrichment in an unregulated control locus (Ins) was not changed. Furthermore, single methylation status of H3K4 stays unchanged (Fig. 5f). H3K4me1 is a mark for transcriptional activation^39^ expected to be independent of the availability of acetyl moieties. Hence, we provide strong evidence for a connection of NAA catabolism and transcriptional regulation via modification of acetylation of activating histone marks in brown adipocytes.

## Discussion

In this study we present evidence for a central role of the metabolite N-acetylaspartate (NAA) in brown adipocyte biology and present a new pathway for the production of cytoplasmic acetyl-CoA. After our previous finding that constitutive overexpression of the NAA producing enzyme Asp-NAT (encoded by Nat8l) enhances the brown adipogenic phenotype^25^, the aim of this study was to functionally characterize the NAA pathway downstream of Asp-NAT.

**Figure 6:**
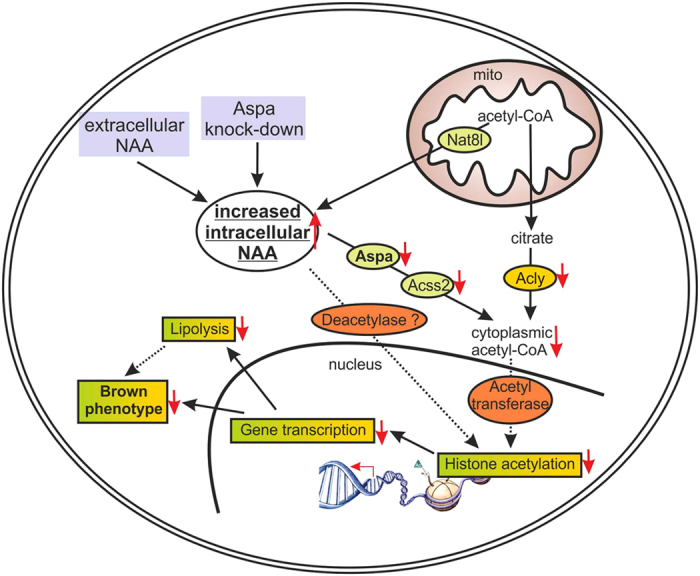
Working model. Working model for NAA pathway impacting on gene transcription via modulation of acetyl-CoA pool and of histone acetylation in brown adipocytes (see text in “Discussion”).

The NAA pathway is well characterized in brain^40^ but only scarcely described in peripheral tissues^22^,^23^,^24^,^25^. Here, we show robust expression of Aspa in mature brown adipocytes and in murine brown adipose tissue. This is also true for other constituents of the NAA pathway (Nat8l^25^, Acss2 (Fig. 4b)). Therefore, the entire pathway (Fig. 6) seems to function in a cell autonomous manner in brown adipocytes. This is surprising considering that in brain the NAA pathway is separated between two cell types. While Asp-NAT synthesizes NAA predominantly in neurons, Aspa catabolizes NAA-mainly in oligodendrocytes^12^. There, Aspa has been reported as a nucleo-cytoplasmic protein^41^, while we find specific cytoplasmic localization in brown adipocytes (cultured and *in vivo*). Further, we detect an accumulation of intracellular NAA upon Aspa knock-down, suggesting a rate-limiting role for Aspa in NAA breakdown in brown adipocytes. This is in accordance with increased NAA levels found in brain of patients with deleterious mutations in the human ASPA gene (Canavan disease)^42^.

One rather surprising outcome is the predominant transcriptional repression upon Aspa knock-down. Mechanistically, we envision the following scenario that could explain this transcriptional repression (Fig. 6): In brown adipocytes, Aspa seems to be essential for funnelling NAA into the cytoplasmic acetyl-CoA pool as we find that Aspa knock-down results in intracellular NAA accumulation and reduced cytoplasmic acetyl-CoA levels. Acetyl-CoA is the sole donor for histone and protein acetylation by lysine acetyltransferases^43^. Therefore, the diminished cytoplasmic acetyl-CoA concentration likely leads to the reduced histone acetylation that we measured for histone H3 and for locus-specific lysine residues (H3K9 and H3K27). The latter histone modifications are known to regulate transcriptional activation^38^,^44^. H3 and H3K9 acetylation have also been associated with Canavan disease in mouse brain white matter^45^ and rat oligodendrocytes^46^, respectively. Our results are also comparable to Wellen and colleagues^47^ who showed that reduced expression of the acetyl-CoA producing enzyme ATP-citrate lyase (Acly) results in an impairment of histone acetylation in several mammalian cell systems. Further, Wellen *et al.* proposed that Acss2 could provide an additional source of acetyl-CoA for histone acetylation reactions^47^. This pathway of acetyl-CoA supply for protein acetylation has also been discussed for NAA-derived acetate in the brain, where Aspa and Acss2 have been found to colocalize^40^. In a recent study, Acss2-derived acetate was shown necessary for Hif-2a acetylation for hypoxia-induced erythropoiesis^48^. Similarly, in brown adipocytes acetylation states of a number of proteins could be dependent on Aspa activity and on the flux through the NAA pathway in general^40^,^49^.

Thus our data corroborate our model (Fig. 6), stating that the NAA pathway acts in parallel to Acly for cytoplasmic acetyl-CoA production, ultimately impacting on histone acetylation and gene transcription. However, we note that in addition to Aspa knock-down, supplementation with exogenous NAA leads to a similar reduction in expression of brown marker and NAA pathway genes. The common denominator in these experimental settings is the increase in intracellular NAA levels. Therefore, an alternative explanation could be that NAA itself directly impacts the activity of (histone) deacetylases resulting in reduced histone acetylation and diminished gene expression. In favour of this hypothesis, we measure unchanged cytoplasmic acetyl-CoA levels in NAA treated iBACs (Fig. S1).

In comparison to our former study^25^, where Nat8l overexpression leads to an increased brown phenotype, it is surprising that treatment with exogenous NAA elicits the opposite outcome. However, while Nat8l overexpression increases Aspa expression^25^ and activity (our unpublished data), NAA treatment reduces Aspa expression. These data argues for a key role of Aspa as NAA flux valve to ultimately determine the phenotype of brown adipocytes.

We did not detect any expression of known brain NAA transporters^12^ in brown adipocytes, but we see a massive increase of intracellular NAA levels when NAA is supplemented to culture medium at a concentration of 10 mM, which corresponds to the physiological concentration measured in brain^12^. This suggests that one or more unknown NAA transporters exist in brown adipocytes. However, we measure a robust expression of two out of three known brain NAA transporters in the small intestine, more specifically in duodenum and ileum (Fig. S2). This supports the observation that NAA is absorbed from dietary sources^30^. Considering the presence of NAA in a number of foods^29^ and the observed effects of NAA on brown adipocytes, it is of great interest to identify BAT-specific NAA transporters.

It has been shown that NAA does not cross the blood brain barrier from the peripheral blood stream to the brain^31^,^32^. However, available data propose that NAA is secreted from the brain because increased NAA levels in urine and plasma of patients with Canavan disease (CD) correlate with high NAA levels in their brains^50^. Until recently, the NAA pathway was chiefly described in brain. Our data clearly revealed that this pathway is highly abundant in brown and white adipocytes and liver, thus the high NAA concentrations found in urine of CD patients might also result from NAA secretion from adipose and other tissues. Interestingly, a number of publications showed that concentrations of urinary NAA as well as the expression levels of Nat8l and Aspa are changed in obesity and/or diabetes^24^,^25^,^26^,^27^,^28^. While we and others showed decreased Nat8l expression^25^ and urinary NAA excretion in ob/ob mice^26^,^28^, aging diabetic rats and diabetic humans have increased urinary NAA concentration^28^. Although the available data are partly inconsistent and need further scrutiny, it argues for a role of the NAA metabolism in obesity and diabetes.

Atgl-mediated lipolysis is essential for the maintenance of functional BAT^35^ by producing ligands for Ppara and Ppard^33^. In our study, we consistently find decreased FFA release independent of the method used to increase intracellular NAA. This could suggest that decreased lipolysis is the primary factor causing the reduced brown phenotype. However, we also see consistent with downregulation of Atgl, decreased triglyceride hydrolase activity in Aspa knock-down cells (Fig. S3), and reduced acetylated histone marks in the Atgl promoter. Together, this suggests that the broad transcriptional repression, rather than reduced lipolysis, is the primary event causing the diminished brown phenotype.

Intense research on BAT biology during the last decade has substantially increased the knowledge about factors influencing BAT recruitment and function. Expansion and activation of BAT as well as “browning” of white adipose depots are promising anti-obesity strategies nowadays^2^,^11^. Here, we show that the NAA pathway and the NAA-catabolizing enzyme Aspa are important to regulate lipolysis and energy homeostasis in brown adipocytes. Hence, modulating the NAA pathway in adipose tissue could be an intriguing approach to influence browning, especially because constituents of the NAA pathway are also robustly expressed in white adipose depots (Nat8l^25^, Aspa (Fig. 2a), Acss2 (biogps.org)).

In accordance with our previous study^25^, we provide further evidence for the existence and biological importance of the NAA pathway in peripheral tissues. Mechanistically, we show that reduced catabolism of NAA after Aspaknock-down leads to significantly reduced cytoplasmic acetyl-CoA levels thereby decreasing histone acetylation and the brown adipocyte phenotype. Our results establish the NAA pathway as working in parallel to the Acly pathway to provide cytoplasmic acetyl-CoA from mitochondrial metabolites. Taken together, these results render the NAA pathway a potential target to develop therapeutic interventions in obesity research.

## Methods

### Cell culture experiments

Immortalized brown adipogenic cells^51^ (iBACs, kind gift of Patrick Seale) were maintained in 4.5 g/l glucose DMEM containing 10% FBS, 50 μg/ml streptomycin, 50 units/ml penicillin/streptomycin, and 20 mM Hepes (all Invitrogen) at 37 °C and 5% CO_2_. For differentiation, medium of confluent cells was supplemented with 0.5 mM 3-isobutyl-1-methylxanthine (IBMX) (Calbiochem), 0.5 μM dexamethasone, 20 nM insulin, 1 nM triiodthyronine, and 125 μM indomethacin (all Sigma). Two days after induction, medium was changed to maintenance medium containing 20 nM insulin and 1 nM T3 and cells were kept in this medium until harvest. For NAA treatments cells were differentiated in presence or absence of indicated concentrations (1, 5, or 10 mM for experiments in Fig. 1b–d, otherwise 10 mM). The pH of media was neutralized after addition of NAA powder (Sigma). For stable knock-down of Aspa, lentiviral shRNA particles and a non-targeting control construct were obtained from Sigma (CSTVRS-TRCN0000098802). Infections of pre-confluent cells (MOI 5) were followed by G418 (1.5 μg/μl, Roth) for seven days selection. Oil red O staining, glycerol and FFA release were performed as described earlier^25^,^52^,^53^. Extracellular FFA was measured after 4 hours pre-incubation in growth medium without FBS containing 2% FFA-free BSA (PAA). Triglyceride hydrolase activity assay was performed as described elsewhere^54^. Primary brown adipocytes were isolated and cultivated as described elsewhere^55^. Briefly, fat depots were digested in DMEM (4.5 g/l glucose) containing 1.5 U/ml collagenase D and 2.4 U/ml dispase II (Roche) for 30 min at 37 °C. Primary cells were filtered through a 100 μm cell strainer and centrifuged at 500 × g for 10 min. Stromal vascular fraction pellets were rinsed and plated on 0.1% gelatine-coated plates.

### Aspa activity assay

Differentiated iBACs (day 7–8 of differentiation) were harvested in homogenization buffer (PBS pH 8.5, 1 mM DTT, 10% glycerol) and sonicated 3× for 5 s. Cell homogenates were centrifuged for 10 min/10000 × g/4 °C and supernatants were collected. An equal amount of 2× reaction buffer (dH_2_O pH 8.5, TrisHCl 100 mM, NaCl 100 mM, CaCl_2_ 5 mM, DTT 0.2 mM, IGEPAL CA-630 (1%, Sigma) 0.5%, N-acetylaspartic acid (Sigma) 40 mM) was added to the samples and a defined volume per sample was collected to provide the same background matrix for the aspartate standard curve. The background matrix was heated at 95 °C for 10 min and subsequently centrifuged for 3 min/16000 × g/4 °C. The background supernatant was collected and all samples and background sample were incubated for 18 h/38 °C/350 rpm in a thermomixer. After incubation, all samples were inactivated at 95 °C for 10 min and subsequently centrifuged for 3 min/16000 × g/4 °C. Supernatants were used for analyzing released aspartate content using the aspartate assay kit (BioVision).

### LC-MS/MS measurement of NAA in iBACs

Cells were washed twice with cold PBS, scraped in 1 ml pre-chilled MeOH, and transferred to 2 ml tubes. Cells were lysed by adding glass beads (~100 μl) and rotating vigorously for 10 min at 4 °C. Proteins were precipitated by incubating 10 min on ice. After centrifugation the clear supernatant was evaporated using speedvac. Samples were analyzed on an ABSCIEX 4000 QTRAP in MRM mode with an Agilent 1100 series capillary LC system. 1 μl of sample was injected onto a polyhydroxyethyl A (PolyLC Inc) capillary HILIC column. Solvent A was 10 mM ammonium acetate in H_2_O, solvent B was 10 mM ammonium acetate in 90% acetonitrile (all Sigma). At a constant flow rate of 10 μl/min, a 7 min linear gradient was employed. The 4000 QTRAP mass spectrometer was equipped with a heated ESI source set to 250 °C, negative polarity, −3500 V. NAA transitions monitored were 174.1 −> 88.0 (CE −20 V) and 174.1 −> 130.0 (CE −15 V). The employed software was Analyst v1.6.1.

## HPLC/HRMS

Cells (cultivated in 6-well plates) were washed once with 10 mM ammonium acetate buffer and washing solution was thoroughly removed. For total metabolomics analysis, plates were stored at −80 °C until extraction. For plate extraction, 110 μl of internal standard mix (Mix of 990 μl ^13^C_2_-acetyl-CoA (120 μM stock in water, Sigma) + 10 μl ^13^C_6_-NAA (10mM stock in water, Sigma)) were added per sample. Extraction procedure was performed according to Ritter *et al.*^56^. Two ml of 75% boiling ethanol were added to each sample immediately after the plate was placed in a water bath (90 °C) and incubated for 2 min. Cooled samples were collected and centrifuged for 10 min/17000 × g at room temperature. Samples were dried using a nitrogen evaporator.

To yield cytosolic metabolites, cells were harvested in 1 ml ice cold 10 mM ammonium acetate buffer (including 1× protease inhibitor cocktail) and 110 μl of internal standard mix (see above) was added per sample. Cells were homogenized by grinding (1 ml douncer, Wheaton, 50 strokes) and centrifuged for 30 min/16000 × g/4 °C. Supernatant was collected and proteins were precipitated overnight using acetone (1/5, sample/acetone, vol/vol). After centrifugation for 20 min/12000 × g/4 °C cytosolic fraction was collected, lyophilized and stored at −80 °C until measurement. Protein content of the pellet was used for normalization.

All samples were resuspended in 100 μl of MilliQ water and centrifuged again. Eight calibration standards containing the same amount of internal standards were prepared. Highest concentration of NAA (80 μM) and acetyl-CoA (72 μM) in water were consecutively diluted in a serial 1:3 dilution.

A Dionex Ultimate 3000 HPLC setup, equipped with an Atlantis T3 C18 analytical column (Waters), was used for compound separation prior to high resolution mass spectrometric detection with an Exactive^TM^ Orbitrap (Thermo Scientific). A reversed-phase ion-pairing HPLC method was used for metabolite separation^57^. A 40 min gradient was applied and 2-propanol and an aqueous phase (5% methanol (*v*/*v*), 10 mM tributylamine, 15 mM acetic acid, pH 4.95) were used as eluents. The injection volume was 10 μl per sample and calibration standard. Negative ionization of metabolites was carried out via heated electrospray ionization prior to mass spectrometric analysis. For the untargeted online detection of analytes, a full scan of all masses between 70 and 1100 m/z with a resolution (R) of 50000 (at *m*/*z* 200) was used.

LC/MS-data acquisition was conducted with Xcalibur software (vers. 2.2 SP1, Thermo Fisher Scientific (Waltham)). Targeted compound analysis for NAA, ^13^C_6_-NAA, acetyl-CoA, and ^13^C_2_-acetyl-CoA were carried out with TraceFinder^TM^ software (version 3.1, Thermo Fisher Scientific (Waltham)). Absolute concentrations were calculated from the peak area ratio of compound and internal standard by a linear calibration function.

### Mouse experiments

Animal procedures were approved by the Austrian Ministry for Science and Research and the experiments were carried out in accordance with the approved guidelines. Male wild-type C57Bl/6J mice (Jackson Laboratories) were kept on regular chow diet in a 12 hours light/dark cycle. Before harvesting the tissue pads of 24–26 weeks old mice they were fasted for 12 hours, followed by re-feeding for 1 hour and then sacrificed. Indicated tissues were dissected, flash frozen in liquid N_2_, and stored at −80 °C until total RNA extraction. For cold exposure experiments C57Bl/6J mice (age 9 months) on chow diet were challenged by cold exposure (4–8 °C) for a period 9 consecutive days. Rectal body temperature was monitored every 30 minutes within the first 12 hours of cold challenge, followed by control measurements every 6–12 hours.

### Western blot analysis

Cell fractionation^25^ and western blot analysis^53^ was performed as described previously. Briefly, 4–12% Novex Bis-Tris gel (Life Technologies) was used with 70 μg protein per lane, followed by blotting to nitrocellulose membrane (PALL). Histones from d7 iBACs were acid extracted according to^58^ and histone pellet of one 10cm petri dish was resuspended in SDS lysis buffer. Antibodies used: α-Aspa (ab112530, Abcam), α-Ucp1 (662045, Millipore), α-β-actin (A5316, Sigma), α-panH3ac (39139, Active Motif), α-totalH3 (4499, Cell signaling). Detection was performed using ECL prime substrate from GE Healthcare. Before reprobing, blots were stripped with Restore WB stripping buffer (Pierce) for 20 min.

### qPCR analyses

Total RNA from cells was isolated using the PeqGOLD total RNA isolation kit (Peqlab). Tissue RNA was isolated with TRIzol reagent (Life Technologies). cDNA was generated using Qiagen QuantiTect RT kit. mRNA expression was assessed using SYBR green real-time PCR on a ABI 7000 instrument as described^53^. Primers used are listed in Supplemental Table 2. Expression values were normalized to 36b4 expression and calculated with an in-house tool^59^ employing the AnalyzerMiner algorithm^60^.

### Mitochondrial respiration measurement

Mature iBACs treated or not with 10 mM NAA were detached with 0.25% trypsin and plated in XF96 polystyrene cell culture microplates (Seahorse Bioscience) at a density of 70000 cells/well. After overnight incubation, cells were washed and incubated for 40 min in unbuffered XF assay medium supplemented with 5.5 mM d-glucose and 1 mM sodium pyruvate at 37 °C in a non-CO_2_ environment. Basal oxygen consumption rate (OCR) was measured using an XF96 extracellular flux analyzer (Seahorse Bioscience), normalized to cell number and calculated as pmol O_2_/min × 7*10^5^ cells.

For Aspa knock-down and ntc control iBACs respiration rate measurement was performed using a Clark electrode (Strathkelvin Instruments). Fully differentiated cells were detached using a cell scraper and subsequently transferred into the MT200A measurement chamber containing 0.3 ml of 100% air saturated respiration buffer (2% BSA, 0.45% D-Glucose, 6 mg pyruvate −545 μl of a 100 mM stock). Oxygen consumption rates were normalized to cell number and calculated as μg O_2_/min/10^6 ^cells.

### Electron microscopy

High pressure freezing with a Leica EM HPM 100 and freeze substitution: cells were grown and differentiated for 7 days on carbon coated sapphire discs. Discs were loaded and frozen using 2000 bar under liquid nitrogen conditions within milliseconds followed by freeze substitution in acetone by adding 2% osmium tetroxide (OsO_4_) and 0.2% UAc (best cryopreservation was obtained with 2% OsO_4_) and 0.2% UAc. The intracellular water was replaced by substitution media. After substitution, the samples were embedded in epoxy resin^61^. Diameters of more than 800 lipid droplets were measured from each Aspa knock-down and control electron micrographs. Mitochondria were measured from 3 replicates (total 99 electron micrographs) and related to total picture area.

### Microarray experiments and functional annotation

Total RNA was isolated with PeqGOLD total RNA isolation kit (Peqlab) according to manufacturer’s recommendations yielding a RIN of 9.5–10 as determined by Agilent 2100 Bioanalyzer. Two-hundred ng of total RNA were prepared for Affymetrix hybridizations on Mouse Gene 2.0 ST Array. Raw array data were normalized using RMA method implemented in CarmaWeb (https://carmaweb.genome.tugraz.at/carma/). Data was deposited in NCBI gene expression omnibus (GEO) with the accession number GSE46495. For DAVID functional annotation, Ensembl Gene IDs of differentially regulated gene lists were submitted to the DAVID website^62^. GO_FAT terms and KEGG pathways were considered significantly enriched if the Benjamini-Hochberg corrected p-value was <0.05. Heatmap was generated with Genesis^63^.

### Chromatin immunoprecipitation (ChIP)

Mature iBACs were cross-linked adding 1% formaldehyde to the medium and shake-incubate for 15 min at RT. Cross-linking was stopped by the addition of 0.125 M glycine for 5 min. Cells were harvested in ice-cold PBS, washed, centrifuged, and flash frozen. The pellet from one 10 cm petri dish was resuspended in 1.5 ml cell lysis buffer (20 mM HEPES/NaOH pH 7.5, 0.25 M Sucrose, 3 mM MgCl2, 0.2% NP-40, 3 mM 2-mercaptoethanol, 1 mM PMSF, protease inhibitor cocktail) and nuclei were extracted by douncing and brief sonication. Pellet was washed twice with cell lysis buffer to remove lipids. Nuclei were lysed in 380 μl ice-cold nuclei lysis buffer (50 mM HEPES/NaOH pH 7.5, 1% SDS, 10 mM EDTA, 1 mM PMSF, protease inhibitor cocktail) by 10 min incubation on ice. Chromatin/DNA mixture was then subjected to ten 20 s bursts using an HD2070 sonicator (Bandlin) at 40% output. Fragmented DNA was checked on an agarose gel to range between 200–800 bp. Fragmented material was mixed 1:10 with IP dilution buffer (50 mM HEPES/NaOH pH 7.5, 155 mM NaCl, 1.1% Triton X-100, 0.11% Na-deoxycholate, 1mM PMSF, protease inhibitor cocktail) and IP was done using precleared Protein G DynaBeads magnetic beads (Life Technologies). Each 5 μg of the following antibodies was used: α-H3K9ac (06–942, Millipore), α-H3K27ac (ab4729, Abcam), α-H3K4me1 (ab8895, Abcam), α-IgG (sc-2027, Santa Cruz). IP material was washed (50 mM Tris, 0.15 M NaCl, 5 mM EDTA, 0.5% NP-40, 1% Triton X-100) six times and eluted in 200 μl buffer (0.1 M NaHCO3, 1% SDS). Immunoprecipitated chromatin and input chromatin were reverse cross-linked and column purified. DNA was subjected to SYBR green qPCR analysis. Measurements were normalized to an 18S rRNA upstream region and related to input measurements to obtain relative enrichment values. Primers used are listed in Supplemental Table 2.

### Statistical analyses

All experiments were performed in triplicates (three different passages) unless indicated otherwise. Bar graph data are shown as means ± SEM. Significant differences between two groups were assessed by two-tailed, unpaired student’s t-test. For ChIP-qPCR experiments a one-sample t-test was used. Significance computation for microarray comparison of control and Aspa knock-down samples was done using a moderate t-test followed by multiple-testing correction (Benjamini-Hochberg). Corrected p-values <0.05 were considered differentially expressed. Also, for DAVID analyses GO terms and KEGG pathways were considered as significantly enriched with Benjamini-Hochberg’s corrected p-value of <0.05.

## Additional Information

**Accession Codes:** The microarray data was deposited in NCBI gene expression omnibus (GEO) with the accession number GSE46495.

**How to cite this article**: Prokesch, A. *et al.* N-acetylaspartate catabolism determines cytosolic acetyl-CoA levels and histone acetylation in brown adipocytes. *Sci. Rep.* **6**, 23723; doi: 10.1038/srep23723 (2016).

## Supplementary Material

Supplementary Figures

Supplementary Table S1
