# Supplementary material for: N-acetylaspartate catabolism determines cytosolic acetyl-CoA levels and histone acetylation in brown adipocytes
Source: Sci Rep. 2016 Apr 5;6:23723. doi: 10.1038/srep23723 (PMC4820693; doi:10.1038/srep23723)
Supplement: Supplementary Figures [file srep23723-s1.pdf]

N-acetylaspartate catabolism determines cytosolic acetyl-CoA levels and histone acetylation in brown adipocytes

Prokesch A., Pelzmann H.J., Pessentheiner A.R., Huber K., Madreiter-Sokolowski C.T., Drougard A., Schittmayer M., Kolb D., Magnes C., Trausinger G., Graier W.F., Birner-Grünberger R., Pospisilik J.A., and Bogner-Strauss J.G.

Supplementary material:

Table S1

Genes upregulated by Aspa knock-down were submitted to DAVID functional annotation[1,2] using GO terms and KEGG pathways. Count shows the number of mapped genes and Benjamini-Hochberg's correction was used for adjusting p-value for multiple testing.

Table S2

Primers used for qPCR analyses.

Name	Assay	Fw Sequence	Rev Sequence
Aspa	qPCR	CCATATGAAGTGAGAAGGGCTC	CCTCAAGAATAAGAGTGCAACC
Ppara	qPCR	CCTGAACATCGAGTGTCGAATATG	GCGAATTGCATTGTGTGACATC
Prdm16	qPCR	TCCACAGCACGGTGAAGCCA	ATCTGCGTCCTGCAGTCGGC
Ucp1	qPCR	ACACCTGCCTCTCTCGGAAA	TAGGCTGCCCAATGAACACT
Nat8l	qPCR	TGTGCATCCGCGAGTTCCGT	GCGGAAAGCCGTGTTGGGGA
Pgc1a	qPCR	TCTCTGGAACTGCAGGCCTAAC	TCAGCTTTGGCGAAGCCTT
ATGL	qPCR	GTCCTTCACCATCCGCTTGTT	CTCTTGGCCCTCATCACCAG
Cidea	qPCR	TGACATTCATGGGATTGCAGAC	GGCCAGTTGTGATGACTAAGAC
Acss1	qPCR	GACACACTAGTTTGGGACACTC	CCCAGATCAAAGCTATGGTCTC
Acss2	qPCR	CTGAGTGGATGAAAGGAGCAAC	CAGGAGTTCACGGTATGTGATC
Acly	qPCR	AGGAAGTGCCACCTCCAACAGT	CGCTCATCACAGATGCTGGTCA
Slc13a1	qPCR	AACCATTGGTGGGCTGACAA	AGTTGAGGCAGCGACAATCA
Slc13a2	qPCR	ACCATGGTGATCTTGCTGCTA	CCTTCCCCAAAGCCAAAGTTC
36b4	qPCR norm.	CAACCCAGCTCTGGAGAAAC	CCAACAGCATATCCCGAATC
Ucp1	ChIP-qPCR	TTTGCATGGATTCCCTCCCC	CCCCCTTCCCATGCATCTAC
Ppara	ChIP-qPCR	AGGGTGACTCTGCCCCAATA	CAGAGTGCCCCCAAAGACAT
Cidea	ChIP-qPCR	CGATGACCCCGAAGAAGGAG	CGCAAGCTACAAGGTCTGGA
Atgl	ChIP-qPCR	TTTGGGGGCTTTAGCCTGAG	TGTCTCCTCCTTCTCCAGGG
Ins	ChIP-qPCR	CTTCAGCCCAGTTGACCAAT	AGGGAGGAGGAAAGCAGAAC

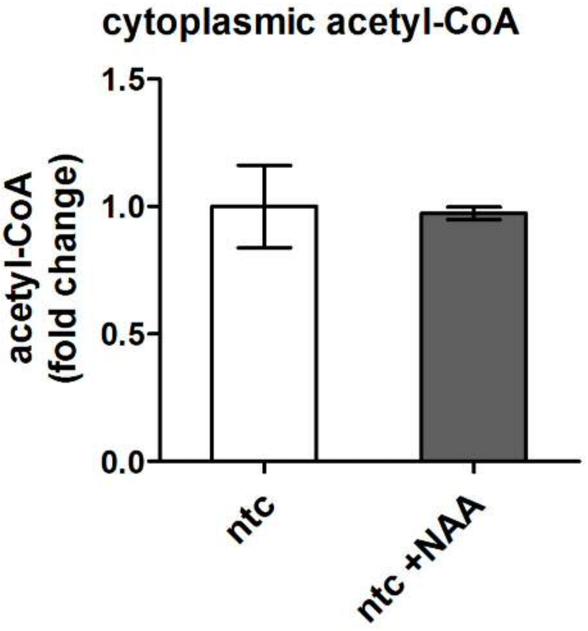

Fig S1

Cytoplasmic acetyl-CoA is not changed in iBACs permanently treated with NAA. iBACs were differentiated in the absence or presence of 10 mM NAA in the differentiation media. Cytoplasmic acetyl-CoA was measured as described in methods (n=6).

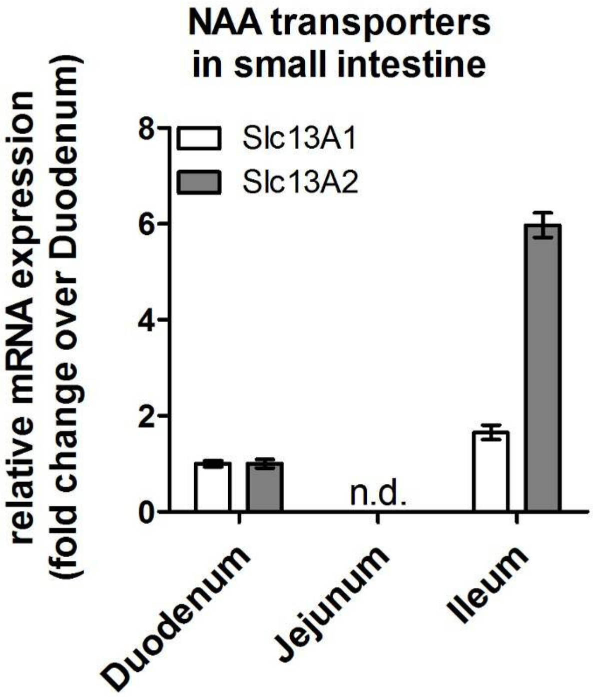

Fig S2

NAA transporters are expressed in the small intestine. Duodenum, jejunum, and ileum of 20-24 week old C57Bl/6 mice (fed state) were harvested and mRNA levels of proposed NAA transporters Slc13A1 and Slc13A2 were measured using qPCR. Expression values were normalized to 18S rRNA. All data are presented as fold change over duodenum (n=3).

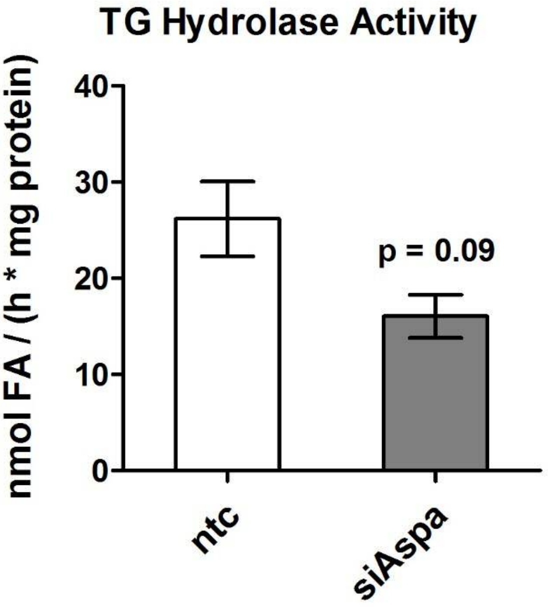

Fig S3

Triglyceride hydrolase activity is reduced upon Aspa knock-down in iBACs. Stably silenced cells were differentiated for seven days and triglyceride hydrolase activity assay was performed as we described elsewhere[3] (n=3). Student´s t-test compared to ntc.

Supplemental Reference List

1. Huang, D. W., Sherman, B. T. & Lempicki, R. A. Systematic and integrative analysis of large gene lists using DAVID bioinformatics resources. *Nat. Protoc.* **4,** 44–57 (2008).

2. Huang, D. W., Sherman, B. T. & Lempicki, R. A. Bioinformatics enrichment tools: paths toward the comprehensive functional analysis of large gene lists. *Nucleic Acids Res.* **37,** 1–13 (2009).

3. Zimmermann, R. *et al.* Fat mobilization in adipose tissue is promoted by adipose triglyceride lipase. *Science* **306,** 1383–1386 (2004).
